# Retraction Note: Knockdown of circRNA_0007534 suppresses the tumorigenesis of cervical cancer via miR-206/GREM1 axis

**DOI:** 10.1186/s12935-022-02827-0

**Published:** 2022-12-15

**Authors:** Qiang Sun, Xiangying Qi, Wenyan Zhang, Xiaoyu Li

**Affiliations:** 1 Department of Obstetrics and Gynecology, Zaozhuang Municipal Hospital, No. 41, Longtou Road, Zaozhuang, 277100 Shandong China; 2 Department of Pharmacology, Shandong Academy of Chinese Medicine, Jinan, Shandong China

**Retraction: Cancer Cell Int (2021) 21:54** 10.1186/s12935-021-01749-7

The Editors-in-Chief have retracted this article. After publication, the authors contacted the journal asking to retract the article because of issues with some experiments, but did not specify those issues. Upon further examination, unexpected similarities were found between western blots presented for different samples, for example panels 4f and 4g. The authors did not provide further comments or raw data. Therefore, the Editors-in-Chief have lost confidence in the data presented here. The authors have not explicitly stated whether they agree to this retraction notice.

